# Effects of temperature, weather, seasons, atmosphere, and climate on the exacerbation of inflammatory bowel diseases: A systematic review and meta-analysis

**DOI:** 10.1371/journal.pone.0279277

**Published:** 2022-12-20

**Authors:** Sun Jae Moon, Yeong Chan Lee, Tae Jun Kim, Kyunga Kim, Hee Jung Son

**Affiliations:** 1 Department of Medicine, Samsung Medical Center, Sungkyunkwan University School of Medicine, Seoul, Republic of Korea; 2 Research Institute for Future Medicine, Samsung Medical Center, Seoul, Republic of Korea; 3 Department of Digital Health, Samsung Advanced Institute for Health Sciences & Technology (SAIHST), Samsung Medical Center, Sungkyunkwan University, Seoul, Republic of Korea; 4 Division of Gastroenterology, Department of Medicine, Samsung Medical Center, Sungkyunkwan University School of Medicine, Seoul, Republic of Korea; 5 Biomedical Statistics Center, Research Institute for Future Medicine, Samsung Medical Center, Seoul, Republic of Korea; 6 Department of Data Convergence and Future Medicine, Sungkyunkwan University School of Medicine, Seoul, Korea; 7 Center for Health Promotion, Samsung Medical Center, Sungkyunkwan University School of Medicine, Seoul, Korea; Kitasato University Kitasato Institute Hospital, JAPAN

## Abstract

**Background:**

Exacerbation of inflammatory bowel disease (IBD) is common. Identification of the exacerbating factors could facilitate interventions for forecastable environmental factors through adjustment of the patient’s daily routine. We assessed the effect of natural environmental factors on the exacerbation of IBD.

**Methods:**

In this systematic review and meta-analysis, studies published from January 1, 1992 to November 3th, 2022 were searched in the MEDLINE, Embase, CINAHL Complete and Cochrane Library databases. We extracted data related to the impact of environmental variations on IBD exacerbation, and performed a meta-analysis of the individual studies’ correlation coefficient χ^2^ converted into Cramér’s V (φc) with 95% confidence intervals (CI).

**Results:**

A total of 7,346 publications were searched, and 20 studies (sample size 248–84,000 cases) were selected. A meta-analysis with seven studies was performed, and the pooled estimate of the correlation (φc) between the seasonal variations and IBD exacerbations among 4806 cases of IBD exacerbation was 0.11 (95% CI 0.07–0.14; I^2^ = 39%; p = 0.13). When divided into subtypes of IBD, the pooled estimate of φc in ulcerative colitis (six studies, n = 2649) was 0.07 (95% CI 0.03–0.11; I^2^ = 3%; p = 0.40) and in Crohn’s disease (three studies, n = 1597) was 0.12 (95% CI 0.07–0.18; I^2^ = 18%; p = 0.30).

**Conclusion:**

There was a significant correlation between IBD exacerbation and seasonal variations, however, it was difficult to synthesize pooled results of other environmental indicators due to the small number of studies and the various types of reported outcome measures. For clinical implications, additional evidence through well-designed follow-up studies is needed.

**Protocol registration number (PROSPERO):**

CRD42022304916.

## Introduction

Inflammatory bowel disease (IBD) is a chronic inflammatory disease of the gastrointestinal tract [1] that has an increasing global prevalence [2]. Following an IBD diagnosis, 1 in 4 patients are hospitalized owing to exacerbation [3]; thus, IBD affects the quality of life of patients [4]. Exacerbation of IBD is related to microbiological, immunological, and environmental factors [5]. Several interventional attempts targeting genetic, microbiological and immunological factors have been undertaken; however, there is no clear evidence that the interventions can alter the exacerbation of IBD [6–9]. Although interventions for environmental factors are limited, they have the advantage of easily inducing changes in the patient’s health behavior depending on the environment and are therefore more cost-effective and could possibly prevent exacerbation without adverse effects.

Studies claiming to show a relationship between the onset of symptoms in established IBD and seasonal variation began to be published in the 1970s [10, 11]. With increasing research on the activity of IBD [12], studies on the relationship between IBD exacerbation and seasonal variations have been published [13]. However, a follow-up study reported that the correlation between IBD exacerbation and seasonal variation was insignificant [14]. In contrast, a large-scale cohort study in Sweden showed a significant correlation of the onset of IBD symptoms with the season or month of birth [15, 16]. Thereafter, some case-series showed that exacerbation was significantly related to a specific month in pediatric or adult UC patients [17], although subsequent studies on IBD exacerbation provided conflicting results [18–27].

A single systematic review of seasonal variation and exacerbation of IBD exists [28], but it is a conference abstract with a different scope because the review did not evaluate temperature and atmosphere besides the seasonal variation. Therefore, we conducted a systematic review and meta-analysis to assess the effect of temperature, weather, seasons, atmosphere, and climate on the exacerbation of IBD.

## Materials and methods

### Search strategy and selection criteria

A systematic review and meta-analysis was conducted and reported according to the PRISMA guidelines [29] and PRISMA checklist presented in the S1 Checklist. The inclusion criteria were as follows: the study should include adult participants diagnosed with IBD who experienced exacerbation; the study should analyze the effects of IBD symptom exacerbation and temperature, weather, season, climate, and atmosphere; studies published in the last 30 years. IBD exacerbation can be defined as the recurrence of symptoms or worsening of the quantified activity index without other secondary causes, or the related medical use such as outpatient visits, hospitalizations or change or increase in IBD medications [30, 31]. Temperature is the quantity of the atmosphere measured with a thermometer, weather refers to the state of the atmosphere, seasons are periods divided by meteorological and climatic features, the atmosphere is the property of gases surrounding the crust, and climate means a change in the weather over a period of more than 30 years [32]. We applied no restriction on the language of publication, and study designs included controlled or observational studies. The exclusion criteria were research on the initial diagnosis of IBD or studies targeting pediatric populations. The pediatric IBD studies were excluded from our review because important factors affecting exacerbation such as nutrition and puberty were different from those of adults [33].

The search was conducted by one author (SJM) using data from the core-databases, MEDLINE, Embase, Cumulative Index Nursing and Allied Health Literature (CINAHL) Complete, and Cochrane Library database on November 3th, 2022. Studies published from January 1st, 1992 to November 3th, 2022 were searched using the publication filter provided by each database. Conference proceedings of the Asia Pacific Digestive Week (APDW) and Australia Gastroenterology Week (AGW), which are not included in the core-database, were manually searched. The search terms included: IBD, “ulcerative colitis” and “Crohn’s disease” and “temperature,” “weather,” “climate” and “atmosphere.” A search query was created using the keyword subjective terms and the truncated form of each term, which were then grouped with the search command. The final search queries were reviewed by the librarian. The entire search results are presented in the S1 Table.

Studies published in foreign languages were translated before review, and for studies where data extraction was difficult owing to the availability of only the abstract, additional data were requested from the respective corresponding authors. Studies with the same author and database name, study area or medical institution name, we contacted corresponding authors to resolving the duplication of sample issue. SJM and HJS performed screening and data extraction independently of each other according to the predefined inclusion criteria. In case of a discrepancy, the same process was repeated until a consensus was reached. Any persistent discrepancies were resolved with the guidance of a third author (KK).

### Data analysis

All authors selected the information list of items to be extracted from each individual study well before the commencement of the review, and a predefined unified form was created using Microsoft Excel (Professional Plus edition). Next, two authors (YCL and SJM) independently performed data extraction. In case of any discrepancies, consensus was reached under the guidance of the third author (HJS). The extracted variables could be divided into publication information, patient-related information, environmental factor related information, and outcome related lists. The patient-related information was further subdivided into sociodemographic and disease-related variables. Sociodemographic extraction variables included sex, age, racial ratio, and income level. Geographically, the latitude, longitude, and altitude of the study city and climate group information according to the Köppen climate classification were extracted [34]. With regard to the disease, (IBD)-related extraction variables, the criteria for diagnosis and subtype of IBD (i.e., UC and CD), and anatomical location, activity, and definition of exacerbation in individual studies were extracted. For the sample size extraction, we distinguished between case-based and person-based enumeration of exacerbation. Moreover, the types of environmental factors and databases were extracted. For the extraction of outcome variables, the methods of statistical analysis, index value (i.e., correlation coefficient or odds ratio), significance, and extractable raw data related to IBD exacerbation and environmental change were all extracted. The quality assessment was independently conducted by two authors (SJM and LYC). Furthermore, for the quality assessment, we used the Newcastle–Ottawa scale (NOS) for cohort and case–control studies [35] and the Joanna Briggs Institute (JBI)’s critical appraisal tools for case-series [36].

A meta-analysis of studies that investigated the relationship between the seasonal variation and the exacerbation of IBD was performed. To facilitate a unified analysis, individual studies were observed and actual exacerbation events were extracted for all the 12 months. The four seasons were defined by the quarterly grouping of months: March–May for spring; June–August for summer; September–November for autumn; and December–February for winter. The seasonal data were obtained from the monthly data and summarized in the contingency tables before conducting the two-tailed chi-square test. Then, the corresponding effect size, Cramér’s V (φc), was converted from the test statistics in each study [37–39]. The pooled estimate and 95% confidence interval (95% CI) for the effect size were obtained by synthesizing the data from the previous studies. Pooled φc is a non-negligible effect if < 0.057, a non-negligible-to-weak effect if 0.058–0.12, a weak-to-moderate effect if 0.12–0.23, a moderate-to-strong effect if 0.23–0.46, and a strong effect if ≥0.46 [38]. A meta-analysis was performed using the random-effects model [40]. Heterogeneity was calculated as an I^2^ statistic with a range of 0–100%, where *I*^2^ values of 1%–49%, 50%–74%, and >75% indicated low, moderate, and high heterogeneity, respectively [41]. In addition, when the number of studies included in the meta-analysis was less than 10, a heterogeneity test was additionally performed with the χ2 square test. If the p-value of this test was less than 0.1, heterogeneity was judged to be significant [42]. Results with <50% heterogeneity were included in [43] further subgroup analyses of the IBD subtype and the climate of the study region. The R metacor package (version 2.1) was used to calculate the pooled estimate of the correlation coefficient with 95% CI and then present the values as forest plots. Publication bias was assessed by drawing funnel plots with odds ratio (OR) after meta-analysis as well as by Egger’s test if more than 10 studies [44]. The review protocol for this research was submitted and registered with the Prospective Register of Systematic Reviews (PROSPERO) prior to study initiation (PROSPERO registration number: CRD42022304916).

## Results

**Fig 1:**
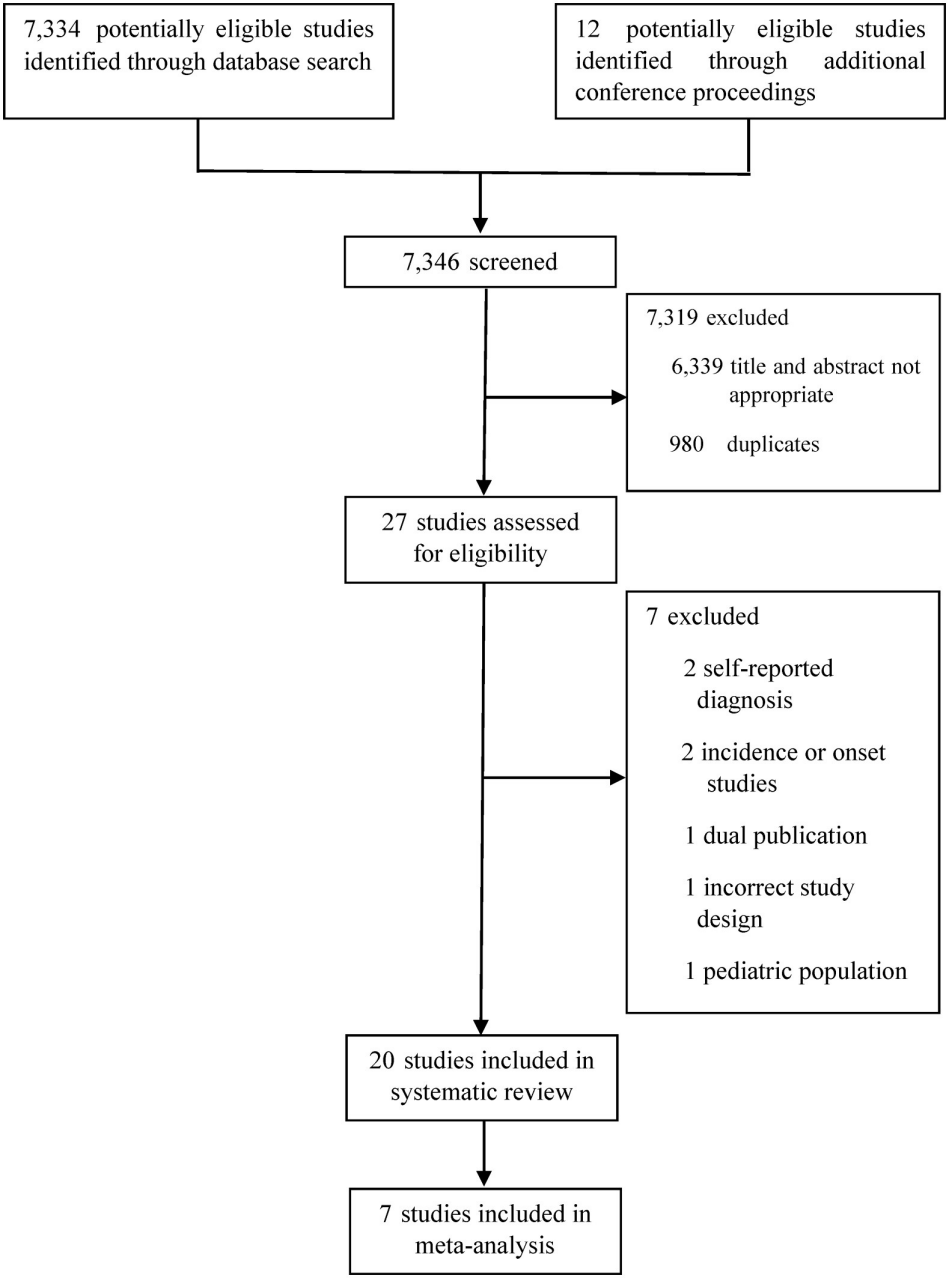
Flowchart of the study selection.

A total of 7,346 publications were searched and screened. Of these, 20 studies met the selection criteria and were finally selected for inclusion in the systematic review (Fig 1). In these 20 studies, the sample size ranged from 248 to 84,000 cases. Of the 20 studies, 3 were on air pollution, 2 on temperature, and the remaining 15 were on seasonal variation. Sociodemographic, geographic characteristics, disease, and exposure characteristics from all 20 studies are summarized in S2 and S3 Tables, and the results of quality assessment are presented in S4 and S5 Tables. Among the studies included, 15 investigated the correlation between seasonal variation and the exacerbation of IBD; moreover, 4 out of 15 studies were conference abstracts and the remainder (11 studies) were journal articles. Furthermore, 3 out of 15 studies were cohort studies and 12 were case-series; 6 out of 15 studies were conducted in Europe and 9 in Asia and the Americas and in particular, most of the studies (9 out of 15 studies) were classified as Köppen Climate Classification Group C (temperate climate) research (Table 1). The number of cases ranged from 164 to 76,608; three studies were UC-only studies and 12 studies were IBD patients (Table 1). In 5 out of 15 studies, IBD exacerbation was defined based on the admission or readmission rate, and 6 studies used an assessment tool for evaluating IBD disease activity or to determine whether a treatment drug was prescribed during the IBD exacerbation; however, three studies used the author’s own definition and one study used both admission and disease activity measurement as the criteria for diagnosing exacerbation (Table 1). A meta-analysis was feasible only for seven studies that reported, predicted, and observed event values over 12 months and used the same methods for statistical analysis (Table 1).

**Table 1:** Key characteristics of seasonal variation studies (*n* = 15).

	Publication type	Study design	Country (city)	Climate group	IBD^a^ sub-type	n (cases)	Definition of exacerbation	Statistics	Study conclusion
Yadav et al. (2019) [45]	Conference abstract	Cohort (retro-prospective) study	Ireland (nationwide)	Group C^d^	IBD	266	Admission	One-way ANOVA^b^	NS^c^
Yadav et al. (2019) [46]	Conference abstract	Cohort (retro-prospective) study	Ireland (Dublin)	Group C	IBD	227	Admission	Chi-square test	Spring and summer
Stein et al. (2016) [47]	Journal	Case-series study	US (nationwide)	Group A^e^ to E^f^	IBD	76,608	Admission	Logistic regression	NS
Peng et al. (2015) [48]	Journal	Case-series study	China (Shanghai)	Group C	IBD	629	Clinical, radiological, endoscopic, and histological features	Chi-square test	Summer
Tinsley et al. (2013) [49]	Conference abstract	Cohort (retro-prospective) study	US (nationwide)	Group A to E	IBD	3360	Readmission	Odds ratio	Autumn
Jung et al. (2013) [50]	Journal	Case-series study	South Korea (Seoul, Gyeonggi)	Group D^g^	IBD	1285	Symptom or additional drug prescription or admission	Chi-square test	Spring
Beaulieu et al. (2009) [26]	Conference abstract	Case-series study	US (Pittsburgh)	Group C	IBD	651	Disease activity measurement	··	Spring
Bai et al. (2009) [25]	Journal	Case-series study	China (Nanchang)	Group C	UC^h^ only	1030	Symptom or additional drug prescription	Chi-square test	Spring and summer
Soncini et al. (2006) [24]	Journal	Case-series study	Italy (nationwide)	Group B^i^ to E	IBD	2856	Admission or disease activity measurement	Logistic regression	NS
Lewis et al. (2004) [23]	Journal	Case-series study	UK (nationwide)	Group C	IBD	4360	Additional drug prescription	Logistic regression	NS
Vergara et al. (1997) [22]	Journal	Case-series study	Spain (Barcelona)	Group C	IBD	560	Disease activity measurement	Chi-square test	Summer
Tezel et al. (1997) [21]	Journal	Case-series study	Turkey (Ankara)	Group C	UC only	164	Disease activity index and endoscopic index	Chi-square test	NS
Karamanolis et al. (1997) [20]	Journal	Case-series study	Greece (Pireaus)	Group B	UC only	248	Symptom or laboratory finding (diarrhea, blood and/or pus in stool, etc.)	Chi-square test	Spring and Autumn
Anderson et al. (1995) [19]	Journal	Case-series study	Canada (Vancouver)	Group C	IBD	892	Symptom assessment by physicians	Chi-square test	Autumn and winter
Sonnenberg et al. (1994) [18]	Journal	Case-series study	US (nationwide)	Group A to E	IBD	28208	Admission	Time-series analysis	Winter

^a^IBD: inflammatory bowel disease.

^b^ANOVA: analysis of variance.

^c^NS: not significant.

^d^Group C: Temperate climate group.

^e^Group A: Tropical climate group.

^f^Group D: Continental climate group.

^g^Group E: Polar climate group.

^h^UC: ulcerative colitis.

^i^Group B: Arid climate group.

**Fig 2:**
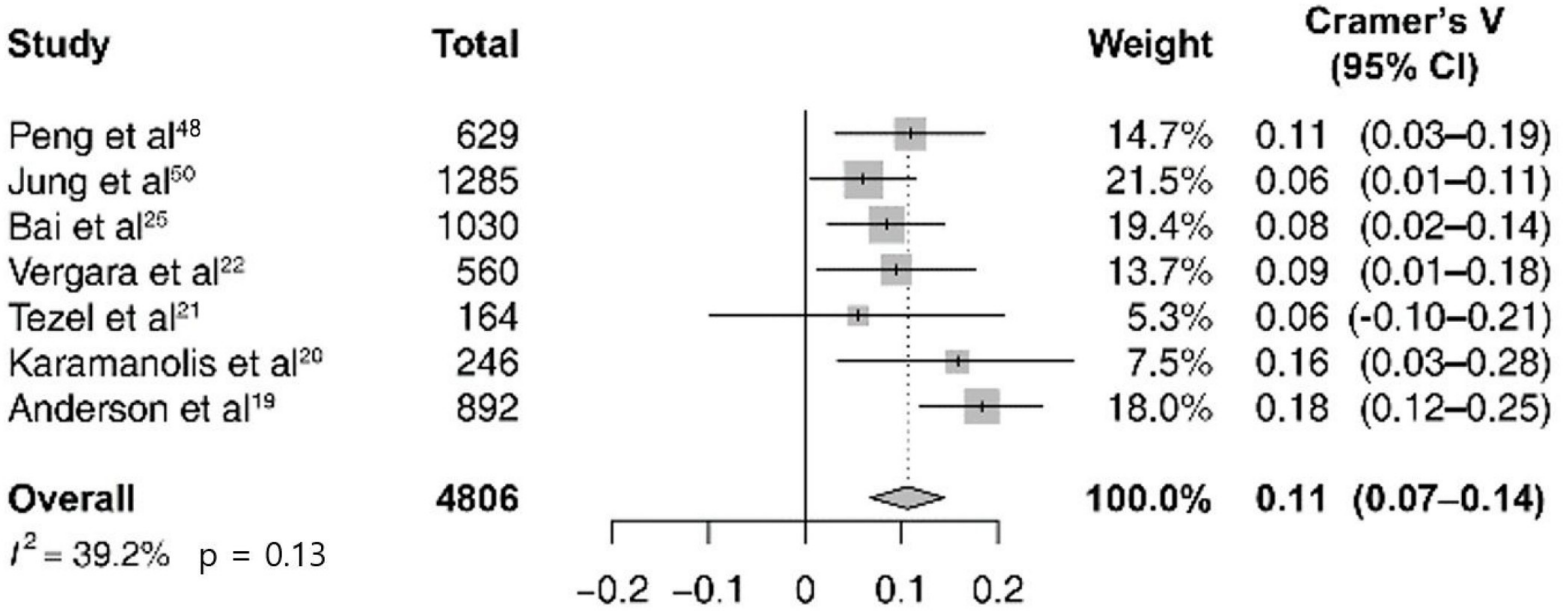
Forest plot of seasonal variation and inflammatory bowel disease exacerbation studies (*n* = 7).

**Fig 3:**
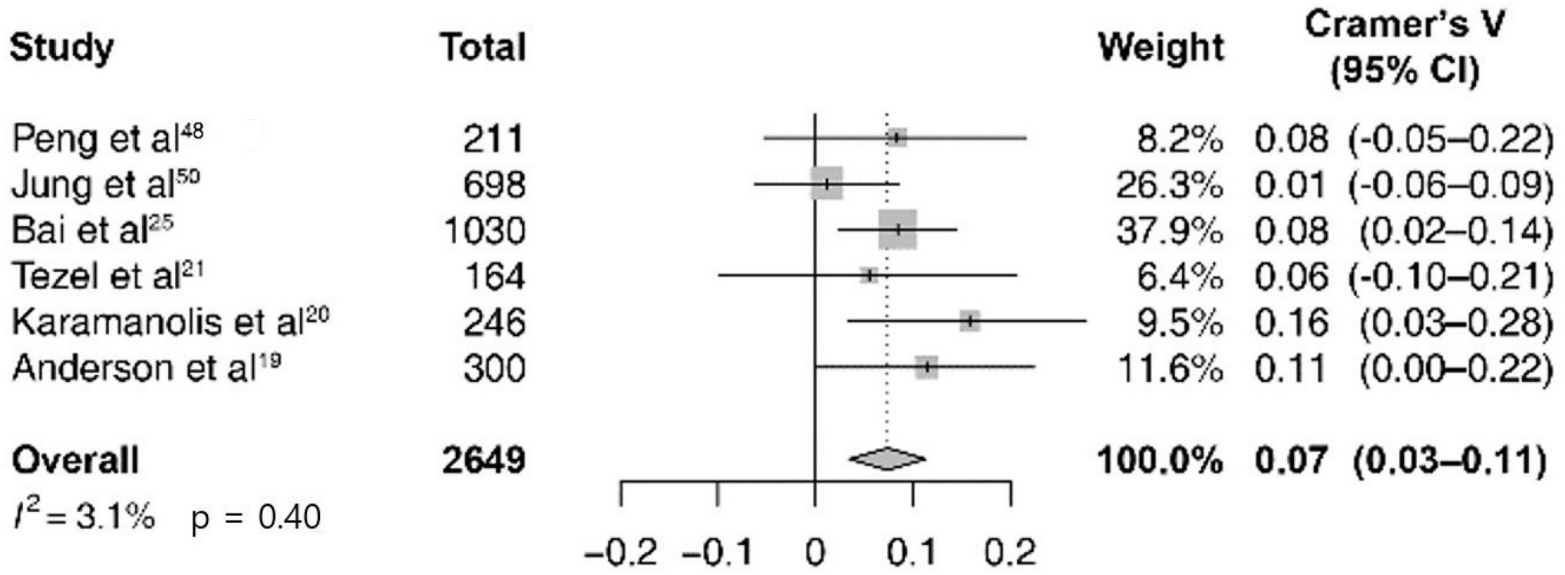
Forest plot of seasonal variation and ulcerative colitis exacerbation studies (*n* = 6).

**Fig 4:**
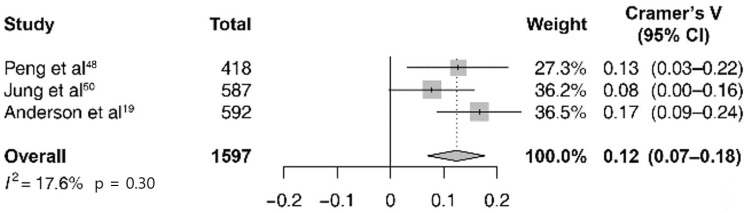
Forest plot of seasonal variation and Crohn’s disease exacerbation studies (*n* = 3).

Among the 4806 cases of total IBD exacerbation in the abovementioned seven studies, the pooled estimate of the correlation between the expected cases and the actual observed ones was 0.11 (95% CI 0.07–0.14; *I*^2^ = 39%; p = 0.13; Fig 2). A subgroup analysis was performed by dividing IBD into the UC and CD subgroups. In six studies, there were 2649 cases of UC exacerbation and in three studies, there were 1597 cases of CD; the pooled estimate of the correlation between the expected cases and the observed cases was 0.07 (95% CI 0.03–0.11; *I*^2^ = 3%; p = 0.40; Fig 3) and 0.12 (95% CI 0.07–0.18; *I*^2^ = 18%; p = 0.30; Fig 4). In all sub-groups, the heterogeneity test was not significant with a p-value of 0.10 or higher. For subgroup analysis according to the Köppen Climate Classification, we identified five temperate climate (Group C) studies and one dry climate (Group B) and one cold climate (Group D) study. The pooled estimates for 3,275 out of five temperate climates was 0.11 (95% CI 0.07–0.14; *I*^2^ = 0%; p = 0.74; S1 Fig), without significant heterogeneity. The publication bias (S2 Fig) and the results of the remaining eight studies that were not included in the meta-analysis (due to the lack of monthly expected and observed values of exacerbation) are not presented.

## Discussion

Our study included a total of 20 studies on the effects of temperature, weather, season, and atmosphere on the exacerbation of IBD [18–27, 45–54], and a meta-analysis of the correlation between the seasonal variation and IBD exacerbation was performed using seven studies; the results showed a non-negligible to weak correlation [18, 20–22, 25, 48, 50]. In the UC subgroup (six studies), seasonal variation showed a non-negligible to weak correlation, whereas in the CD subgroup (three studies), seasonal variation showed a weak positive correlation with the exacerbation of IBD. In addition, the temperate climates subgroup (five studies) showed a non-negligible to weak, but significant positive correlation. There was a low level of heterogeneity between the studies in both overall and subgroup analyses. Although the meta-analysis of three studies on air pollution was not performed [27, 51, 52], all three of the studies reported a significant positive correlation between air pollutants and exacerbation of IBD.

When examining the studies that were not included in the meta-analysis, it seems that IBD exacerbation tended to occur slightly more frequently in warmer seasons. First, among the ten studies that reported a significant correlation between seasonal variation and IBD exacerbation, six reported a significant correlation with spring or summer [22, 25, 26, 46, 48, 50], and the remaining three reported a correlation with winter or autumn [18, 19, 49]; in one study, both spring and autumn were reported (Table 1) [20]. Second, two studies that reported the relationship between short-term extreme temperature change and IBD exacerbation support our hypothesis that warmer seasons were associated with more IBD exacerbations. One study reported that a cold spell was not significantly associated with the exacerbation of IBD [53], whereas another study reported a significant association between a heat wave and IBD exacerbation [54]. However, it is difficult to obtain conclusive results by generalizing seasonality and IBD exacerbation, as a quantitative analysis based on meta-analysis was unavailable.

The strength of this study is the length of the searched publication timeline (30 years) that was sufficient to consider the effect of the natural environment. The studies included in the meta-analysis had low overall heterogeneity and publication bias, and the reproducibility was high because we used the monthly data of the studies as the raw data. However, a limitation of our meta-analysis is that the results only presented the relevant intensity of significance but could not clarify which season was more relevant. In other words, there were only two studies that were presented as comparative statistics (odds ratio) between specific seasons [23, 47], so it was difficult to merge them with a meta-analysis. Another limitation of our study is that with regard to short-term temperature changes and air pollution other than seasonal variations, the number of studies reporting these characteristics was relatively small; therefore, a quantitative evidence synthesis could not be presented by meta-analysis. Cohort studies (three studies) were published only as conference abstracts, and a meta-analysis could not be performed owing to the lack of raw data for meta-analysis. Furthermore, only 6 out of 20 studies reported information on the duration of IBD morbidity, location of lesions, or medications administered [20–24, 50]. Moreover, 7 out of 20 studies did not report the number of patients who experienced IBD exacerbations [18, 20, 26, 27, 49, 51, 52], and 6 of the 11 studies reported different numbers of patients and cases of exacerbations [19, 21, 22, 25, 48, 50]. The abovementioned factors might have resulted in the underestimation or overestimation of the pooled estimate results of the meta-analysis. In addition, the method used for defining IBD exacerbation varied across studies; specifically, 7 out of 20 studies did not use a standard definition (i.e., cutoff for IBD activity measurements or inclusion criteria) (S4 Table). Among the included studies, there was one study in which the outpatient visit was defined as exacerbation [52]. There is a possibility that the outpatient visit may be different from the actual exacerbation time, so if other indicators such as disease activity are not considered together, the risk of misclassification may increase, and interpretation should be careful. This concern emerged from the lack of a clear-cut definition of IBD exacerbation, although attempts were made to reach a consensus on the exacerbation of UC [55], and may have caused potential bias in the individual studies. However, in view of the increasing importance of real-world evidence, this systematic review, which identifies the diversity of definitions of exacerbation in individual studies that were conducted in the clinical setting, is still meaningful.

We found that many studies included in this systematic review did not take into account that the seasonality of exacerbation could be affected by the seasonal habit or culture environment of each country. Only one study considered that exacerbation in summer was reduced since many holidays or vacations in the summer period as a country’s (Greece) characteristic, and the stress of IBD patients may be low [20]. According to the data from the Organization for Economic Co-operation and Development (OECD), among the countries included in this systematic review, European countries such as the UK, Spain, Italy, and Greece took paid leave of 20 days or more, but Canada, the United States, etc. North America and Asia, such as Japan and South Korea receive less than 20 days of paid leave [56]. As such, the habits, culture, and environment affected by the seasons, including the working environment, are different for each country, and this may affect the seasonal nature of exacerbation by acting differently as physical or psychiatric stress for IBD patients, and further research is needed. Another distinctive feature is that the countries and cities in all 20 published studies were located above the Tropic of Cancer in the Northern Hemisphere, especially at 30–50° latitude. This phenomenon of publications originating in the Northern Hemisphere could be considered a potential publication bias. According to the recent Global Burden of IBD study [57], given the increasing prevalence of IBD in all countries in the Southern Hemisphere, it will be necessary to encourage and include publications from countries in the Southern Hemisphere.

In conclusion, this systematic review and meta-analysis showed a significant correlation between the exacerbation of IBD and seasonal variation. However, owing to the small number of included studies and the weak power of the pooled estimate value, the results should be cautiously interpreted. To support the preliminary findings of this research, high-quality, well-designed studies should be conducted.

## Supporting information

S1 Fig Forest plot of seasonal variation and IBD exacerbation of temperate climate studies (seven studies). (TIF)

S2 Fig Funnel plot of seven studies. (TIF)

S1 Table Search queries. (DOCX)

S2 Table Sociodemographic and geographic characteristics of 20 studies. (DOCX)

S3 Table Disease and exposure variable characteristics in 20 studies. (DOCX)

S4 Table Quality assessment of 17 case-series studies. (DOCX)

S5 Table Quality assessment of three cohort studies. (DOCX)

S1 Checklist PRISMA checklist. (PDF)
