# Supplementary figures and images for: Effects of temperature, weather, seasons, atmosphere, and climate on the exacerbation of inflammatory bowel diseases: A systematic review and meta-analysis
Source: PLoS One. 2022 Dec 20;17(12):e0279277. doi: 10.1371/journal.pone.0279277 (PMC9767326; doi:10.1371/journal.pone.0279277)

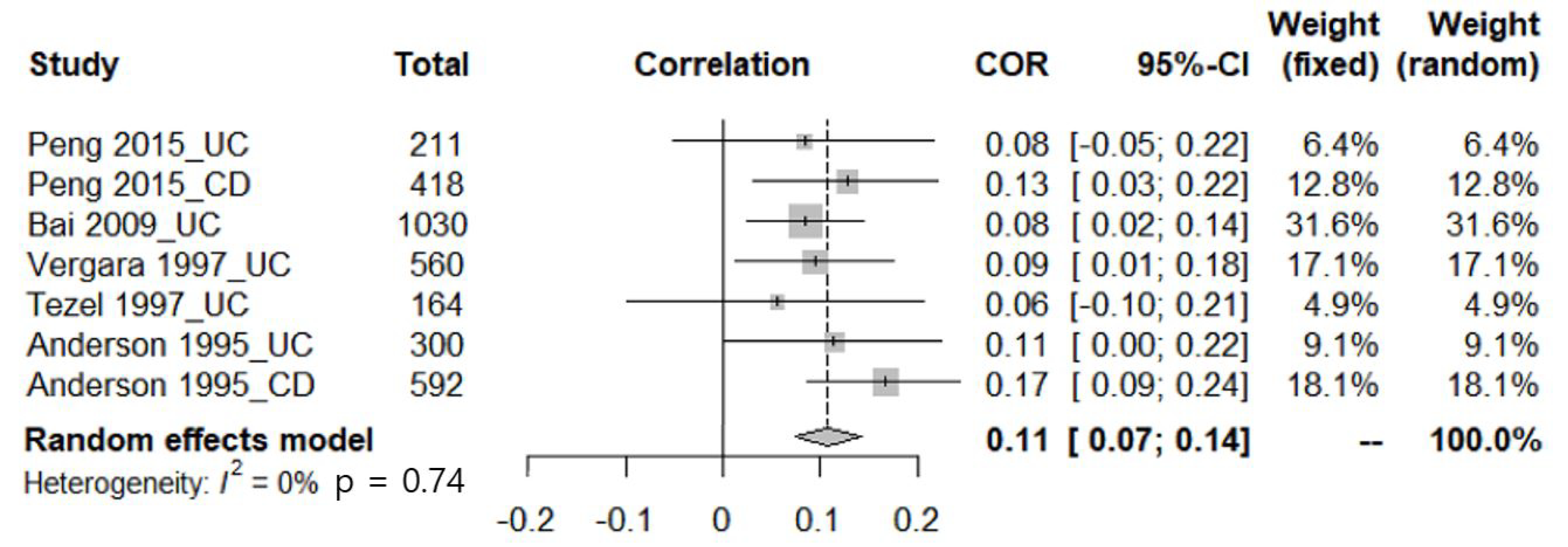

Supplement: S1 Fig — (TIF) [file pone.0279277.s001.tif]

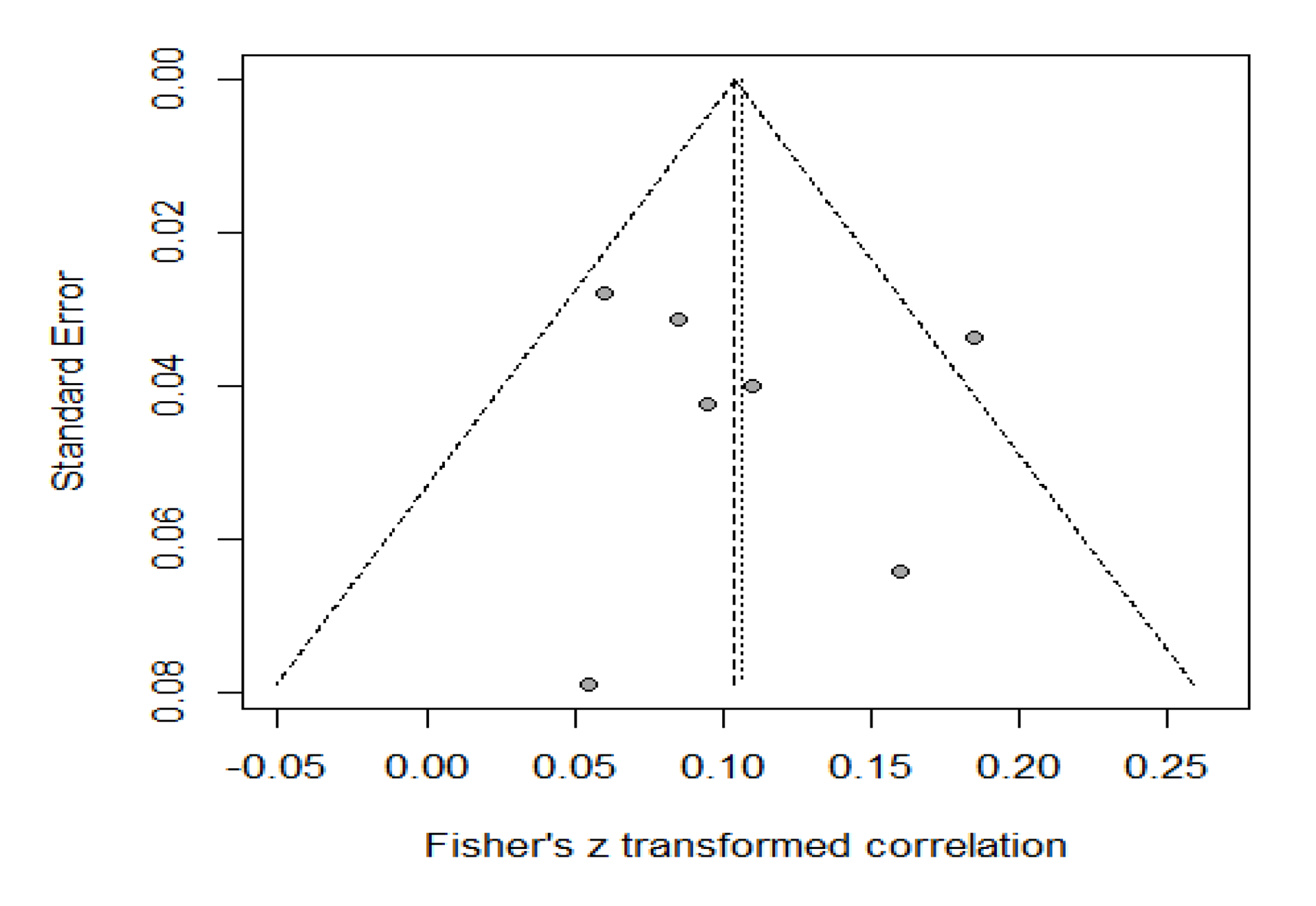

Supplement: S2 Fig — (TIF) [file pone.0279277.s002.tif]
