# Supplementary material for: Effects of temperature, weather, seasons, atmosphere, and climate on the exacerbation of inflammatory bowel diseases: A systematic review and meta-analysis
Source: PLoS One. 2022 Dec 20;17(12):e0279277. doi: 10.1371/journal.pone.0279277 (PMC9767326; doi:10.1371/journal.pone.0279277)
Supplement: S1 Table — (DOCX) [file pone.0279277.s003.docx]

**S1 Table. Search Queries.**

| Database | **Embase & MEDLINE Complete (2022.11.03 GMT 13:05)** |  |
| --- | --- | --- |
| #1 | 'season'/mj OR 'temperature'/mj OR 'weather'/mj OR 'climate'/mj OR 'air pollution'/mj OR season* OR temperature* OR aerobiolog* OR weather* OR climat* OR forecast* OR aerial-pollut* OR aerogenic-pollut* OR air-contaminat* OR air-pollut* OR air-born* OR atmosphere* OR 'degrees celsius' OR (degrees AND ('celsius'/exp OR celsius)) | 1,621,398 |
| #2 | 'inflammatory bowel disease'/exp OR inflammatory-bowel* OR Crohn* OR ulcerative* | 241,236 |
| #3 | #1 AND #2 | 2,293 |
| #4 | #1 AND #2 AND [1992-2022]py | 2,142 |
| Database | **CINAHL Complete (2022.11.03 GMT 08:18)** |  |
| #1 | (MM "Seasons") OR (MM "Temperature") OR (MM "Weather") OR (MM "Climate") OR (MM "Air Pollution") OR season* OR temperature* OR aerobiolog* OR weather* OR climat* OR forecast* OR aerial-pollut* OR aerogenic-pollut* OR air-contaminat* OR air-pollut* OR air-born* OR atmosphere* OR Celsius* | 363,206 |
| #2 | (MM "Inflammatory Bowel Diseases") OR inflammatory-bowel* OR Crohn* OR ulcerative* | 42,240 |
| #3 | #1 AND #2 | 5,063 |
| #4 | #1 AND #2 AND [1992-2022]py | 5,025 |
| Database | **Cochrane Library database (2022. 11.03 GMT 07:22)** |  |
| #1 | MeSH descriptor: Seasons OR MeSH descriptor: Temperature OR MeSH descriptor: Weather OR MeSH descriptor: Climate OR MeSH descriptor: Atmosphere OR season* OR temperature* OR aerobiolog* OR weather* OR climat* OR forecast* OR aerial-pollut* OR aerogenic-pollut* OR air-contaminat* OR air-pollut* OR air-born* OR atmosphere* OR Celsius* | 43,487 |
| #2 | MeSH descriptor: Inflammatory Bowel Diseases OR inflammatory-bowel* OR Crohn* OR ulcerative* | 12,316 |
| #3 | #1 AND #2 | 167 |
| #4 | #1 AND #2 AND [1992-2022]py | 167 |
