# Supplementary material for: Effects of temperature, weather, seasons, atmosphere, and climate on the exacerbation of inflammatory bowel diseases: A systematic review and meta-analysis
Source: PLoS One. 2022 Dec 20;17(12):e0279277. doi: 10.1371/journal.pone.0279277 (PMC9767326; doi:10.1371/journal.pone.0279277)
Supplement: S2 Table — (DOCX) [file pone.0279277.s004.docx]

**S2 Table. Sociodemographic and Geographic Characteristics of 20 Studies.**

|  | **Sex (female, %)** | **Mean age (years)** | **Country** | **Continent** | **City** | **Latitude (degree)** | **Climate group** | **Altitude (meter)** |
| --- | --- | --- | --- | --- | --- | --- | --- | --- |
| Ding et al. (2022) | ·· | ·· | China | Asia | Hefei | 32 | Group C | 37 |
| Duan et al. (2021) | 49∙74% | ·· | China | Asia | Beijing | 40 | Group D | 44 |
| Yadav_a et al. (2019) | 54∙00% | 44∙73 | Ireland | Europe | National-wide | 53 | Group C | 0–1039 |
| Yadav_b et al. (2019) | 55∙95% | 45∙37 | Ireland | Europe | Dublin | 53 | Group C | 20 |
| Manser et al. (2017) | 50∙00% | 47∙90 | Switzerland | Europe | Zurich | 47 | Group C | 408 |
| Stein et al. (2016) | ·· | 44∙46 | US | America | National- wide | 40∙20 | Group A to E | 0–4000 |
| Peng et al. (2015) | 40∙95% | 39∙80 | China | Asia | Shanghai | 31 | Group C | 4 |
| Tinsley et al. (2013) | ·· | ·· | US | America | National-wide | 40∙20 | Group A to E | 0–4000 |
| Manser et al. (2013) | ·· | ·· | Switzerland | Europe | Zurich | 47 | Group C | 408 |
| Jung et al. (2013) | 34∙94% | 35∙48 | South Korea | Asia | Seoul, Gyeonggi | 37 | Group D | 38 |
| Ananthakrishnan et al. (2011) | ·· | ·· | US | America | Wisconsin | 44 | Group D | 176–548 |
| Beaulieu et al. (2009) | 52∙00% | 54∙50 | US | America | Pittsburgh | 40∙44 | Group C | 373 |
| Bai et al. (2009) | ·· | ·· | China | Asia | Nanchang | 32 | Group C | 37 |
| Soncini et al. (2006) | 50∙90% | 42∙57 | Italy | Europe | National- wide | 41∙82 | Group B to E | 100–550 |
| Lewis et al. (2004) | 54∙54% | 45∙41 | UK | Europe | National- wide | 54 | Group C | 162–1345 |
| Vergara et al. (1997) | 46∙54% | 40∙37 | Spain | Europe | Barcelona | 41 | Group C | 12 |
| Tezel et al. (1997) | 46∙80% | 40∙00 | Turkey | Asia | Ankara | 40 | Group C | 938 |
| Karamanolis et al. (1997) | 40∙42% | 48∙70 | Greece | Europe | Pireaus | 38 | Group B | 2 |
| Anderson et al. (1995) | 53∙00% | 40∙00 | Canada | America | Vancouver | 49 | Group C | 0 |
| Sonnenberg et al. (1994) | 65∙00% | ·· | US | America | National-wide | 40∙20 | Group A to E | 0–4000 |
