# Supplementary material for: Effects of temperature, weather, seasons, atmosphere, and climate on the exacerbation of inflammatory bowel diseases: A systematic review and meta-analysis
Source: PLoS One. 2022 Dec 20;17(12):e0279277. doi: 10.1371/journal.pone.0279277 (PMC9767326; doi:10.1371/journal.pone.0279277)
Supplement: S3 Table — (DOCX) [file pone.0279277.s005.docx]

**S3 Table. Disease and Exposure Variable Characteristics in 20 Studies.**

|  | **IBD subtype** | **Diagnostic criteria of IBD (Y/N)** | **Exposure variable** | **Exacerbation definition** | **n (persons)** | **n (CD)** | **n (UC)** | **N (cases)** | **N (CD)** | **N (UC)** |
| --- | --- | --- | --- | --- | --- | --- | --- | --- | --- | --- |
| Ding et al. (2022) | CD and UC | Y | Air pollution | Outpatient visits | 886 | 573 | 313 | 886 | 573 | 313 |
| Duan et al. (2021) | UC only | Y | Air pollution | Outpatient visits | ·· | ·· | ·· | 84,000 | 0 | 84,000 |
| Yadav_a et al. (2019) | CD and UC | N | Season | Admission | 266 | 168 | 98 | ·· | ·· | ·· |
| Yadav_b et al. (2019) | CD and UC | N | Season | Admission | 227 | 142 | 85 | ·· | ·· | ·· |
| Manser et al. (2017) | CD and UC | Y | Cold temperature | Admission | 738 | 418 | 320 | ·· | ·· | ·· |
| Stein et al. (2016) | CD and UC | Y | Season, geographic variation, UV | Admission | ·· | ·· | ·· | 220,103 | 143,495 | 76,608 |
| Peng et al. (2015) | CD and UC | N | Season | Clinical, radiological, endoscopic, and histological features | 901 | 332 | 569 | 629 | 418 | 211 |
| Tinsley et al. (2013) | CD and UC | N | Season | Re-admission | ·· | ·· | ·· | 3360 | 1303 | 2057 |
| Manser et al. (2013) | CD and UC | Y | Heat wave | Admission | 738 | ·· | ·· | ·· | ·· | ·· |
| Jung et al. (2013) | CD and UC | Y | Season | Symptom or additional drug prescription or admission | 727 | 316 | 411 | 1285 | 587 | 698 |
| Ananthakrishnan et al. (2011) | CD and UC | Y | Air pollution | Admission | ·· | ·· | ·· | 3890 | 2537 | 1353 |
| Beaulieu et al. (2009) | CD and UC | N | Season | Disease activity measurement | ·· | ·· | ·· | 651 | 501 | 150 |
| Bai et al. (2009) | UC only | Y | Season | Symptom or additional drug prescription | 409 | 0 | 409 | 1030 | 0 | 1030 |
| Soncini et al. (2006) | CD and UC | Y | Season | Admission or disease activity measurement | 2856 | 1541 | 1315 | ·· | ·· | ·· |
| Lewis et al. (2004) | CD and UC | Y | Season | Additional drug prescription | 4360 | 1587 | 2773 | ·· | ·· | ·· |
| Vergara et al. (1997) | CD and UC | N | Season | Disease activity measurement | 255 | 114 | 141 | 560 | 278 | 282 |
| Tezel et al. (1997) | UC only | N | Season | Disease activity index and endoscopic index | ·· | ·· | ·· | 164 | 0 | 164 |
| Karamanolis et al. (1997) | UC only | N | Season | Symptom or laboratory finding (diarrhea, blood and/or pus in stool etc.) | ·· | ·· | ·· | 248 | 0 | 248 |
| Anderson et al. (1995) | CD and UC | N | Season | Symptom assessment by physicians | 226 | 139 | 87 | 892 | 592 | 300 |
| Sonnenberg et al. (1994) | CD and UC | Y | Season | Admission | ·· | ·· | ·· | 28208 | 13516 | 14692 |
