# Supplementary material for: Effects of temperature, weather, seasons, atmosphere, and climate on the exacerbation of inflammatory bowel diseases: A systematic review and meta-analysis
Source: PLoS One. 2022 Dec 20;17(12):e0279277. doi: 10.1371/journal.pone.0279277 (PMC9767326; doi:10.1371/journal.pone.0279277)
Supplement: S4 Table — (DOCX) [file pone.0279277.s006.docx]

**S4 Table. Quality Assessment of 17 Case-Series Studies.**

|  | **Inclusion criteria** | **Condition measurement** | **Method validity** | **Consecutiveness** | **Inclusion completeness** | **Reporting demographics clearness** | **Reporting participants clearness** | **Outcome reporting clearness** | **Reporting presenting site/clinic clearness** | **Analysis appropriateness** |
| --- | --- | --- | --- | --- | --- | --- | --- | --- | --- | --- |
| Ding et al. (2022) | Yes | Yes | Unclear | Yes | Unclear | Yes | Unclear | Yes | Yes | Unclear |
| Duan et al. (2021) | Yes | Yes | Unclear | Yes | Unclear | Yes | Unclear | Yes | Yes | Unclear |
| Manser et al. (2017) | Yes | Yes | Yes | Yes | Unclear | Yes | Unclear | Yes | Yes | Unclear |
| Stein et al. (2016) | Yes | Yes | No | Yes | Yes | Yes | Unclear | Yes | Yes | Unclear |
| Peng et al. (2015) | Unclear | Unclear | Unclear | Yes | Unclear | Yes | Unclear | Yes | Yes | No |
| Manser et al. (2013) | Yes | Yes | Yes | Yes | Unclear | Yes | Unclear | Yes | Yes | Unclear |
| Jung et al. (2013) | Yes | Yes | Yes | Yes | Unclear | Yes | Yes | Yes | Yes | No |
| Ananthakrishnan et al. (2011) | Yes | Yes | Yes | Yes | Unclear | Yes | Unclear | Yes | Yes | Unclear |
| Beaulieu et al. (2009) | Unclear | Unclear | Unclear | Yes | Unclear | Yes | Unclear | Unclear | Yes | Unclear |
| Bai et al. (2009) | Yes | Yes | Unclear | Yes | Unclear | Unclear | Unclear | Yes | Yes | No |
| Soncini et al. (2006) | Yes | Yes | Yes | Yes | Unclear | Yes | Yes | Yes | Yes | Unclear |
| Lewis et al. (2004) | Yes | Yes | Yes | Yes | Unclear | Yes | Yes | Yes | Yes | Unclear |
| Vergara et al. (1997) | Unclear | Yes | Yes | Yes | Unclear | Yes | Yes | Yes | Yes | No |
| Tezel et al. (1997) | Unclear | Yes | Yes | Yes | Unclear | Yes | Yes | Yes | Yes | Unclear |
| Karamanolis et al. (1997) | Unclear | Unclear | Unclear | Yes | Unclear | Yes | Yes | Yes | Yes | Unclear |
| Anderson et al. (1995) | Unclear | Unclear | Unclear | Yes | Unclear | Yes | Unclear | Yes | Yes | No |
| Sonnenberg et al. (1994) | Yes | Yes | Yes | Yes | Unclear | Yes | Unclear | Yes | Yes | Unclear |
