# Supplementary material for: Effects of temperature, weather, seasons, atmosphere, and climate on the exacerbation of inflammatory bowel diseases: A systematic review and meta-analysis
Source: PLoS One. 2022 Dec 20;17(12):e0279277. doi: 10.1371/journal.pone.0279277 (PMC9767326; doi:10.1371/journal.pone.0279277)
Supplement: S5 Table — (DOCX) [file pone.0279277.s007.docx]

**S5 Table. Quality Assessment of Three Cohort Studies.**

|  | **Selection** |  |  |  | **Comparability** | **Outcome** |  |  | **Total** |
| --- | --- | --- | --- | --- | --- | --- | --- | --- | --- |
|  | **Representativeness of the exposed cohort** | **Selection of the unexposed cohort** | **Ascertainment of exposure** | **Demonstration of outcome of interest** | **Comparability of cohorts based on study design** | **Outcome assessment** | **Was follow-up long enough for outcomes to occur** | **Adequacy of follow-up of cohorts** |  |
| Yadav_a et al. (2019) | 0 | 0 | 1 | 0 | 0 | 1 | 1 | 0 | 3 |
| Yadav_b et al. (2019) | 0 | 0 | 1 | 0 | 0 | 1 | 1 | 0 | 3 |
| Tinsley et al. (2013) | 0 | 0 | 1 | 0 | 0 | 1 | 1 | 0 | 3 |
